# The evolution of post-infection mortality

**DOI:** 10.1098/rspb.2024.1854

**Published:** 2024-11-20

**Authors:** Chadi M. Saad-Roy, Andy White, Mike Boots

**Affiliations:** ^1^ Miller Institute for Basic Research in Science, University of California, Berkeley, CA, USA; ^2^ Department of Integrative Biology, University of California, Berkeley, CA, USA; ^3^ Maxwell Institute for Mathematical Sciences and Department of Mathematics, Heriot-Watt University, Edinburgh, UK; ^4^ Department of Biosciences, University of Exeter, Penryn, UK

**Keywords:** post-infection mortality, pathogen evolution, eco-evolutionary model

## Abstract

COVID-19 infections have underlined that there can be substantial impacts on health after recovery, including elevated mortality. While such post-infection mortality (PIM) is clearly widespread, we do not yet have any understanding of its evolutionary dynamics. To address this gap, we use an eco-evolutionary model to determine conditions where PIM is evolutionarily favoured. Importantly, from a pathogen perspective, there are two potential ‘resources’: never-infected susceptibles and previously infected susceptibles (provided some reinfection is possible), and PIM only occurs in the latter. A key insight is that unlike classic virulence (i.e. during-infection mortality, DIM) PIM is neutral and not selected against in the absence of other trade-offs. However, PIM modulates characteristics of endemicity, and may also vary with other pathogen-specific components. If PIM is only correlated with transmission, recovery or DIM, it simply acts to modulate their impacts on the evolutionary outcome. On the other hand, if PIM trades off with the relative susceptibility to reinfection, there are important evolutionary implications that contrast with DIM. We find settings where a susceptibility–mortality trade-off (i.e. an increase in mortality leads to higher relative susceptibility to reinfection) can select against DIM but favour PIM. This provides a potential explanation for the ubiquity of PIM. Overall, our work illustrates that PIM can readily evolve in certain settings and highlights the importance of considering different sources of mortality.

## 1. Introduction

As highlighted by the ongoing SARS-CoV-2 pandemic (e.g. [1]), infection by pathogens may lead to an increase in mortality after a host recovers. Such delayed mortality in response to pathogen infection could emerge through a variety of mechanisms. For instance, post-infection mortality (PIM) could be caused by damage encountered during active infection, which gives rise to long-term complications that arise much later after infection. For SARS-CoV-2, long-term complications may include cardiovascular [2] or neurological [3] issues (see also Davis *et al*. [4] for a review of ‘long COVID’). More generally, a number of pathogens leave symptoms post-infection (see Choutka *et al*. [5] for a review of this phenomenon).

While PIM may be a common phenomenon, understanding the ensuing impacts on infectious disease dynamics is an active area of research. To elucidate the potential epidemiological impacts of PIM, Saad-Roy *et al*. [6] used a simple epidemiological model that separates never-infected susceptibles from those previously infected (and recovered). Due to the interaction between PIM and this previously infected susceptible pool, they showed that PIM can trigger epidemic cycles and lead to complex dynamics in a seasonal environment. Additionally, they found that robust host immunity is (intuitively) stabilizing, whereas mortality during active infection can trigger periodicity in the presence of weak PIM. As such, we know that PIM is a widespread phenomenon with important epidemiological consequences, but we have little understanding of its evolutionary dynamics.

A key question in infectious disease epidemiology is to understand the drivers of pathogen evolution. A large body of literature has examined the evolution of virulence, i.e. during-infection mortality (DIM), from theoretical and empirical perspectives (e.g. [7–12]). In addition to virulence, there are a variety of other pathogen traits, such as recovery, invasion, and progression, and these may all be shaped by evolution. In particular, multiple studies have examined pathogen life-history theory under other trade-offs (e.g. transmission–recovery [13], invasion–persistence [14] or progression–transmission [15–17]).

An influential article by Day [18] introduced the concept of ‘timing’ of transmission and mortality on selection for changes in virulence. Importantly, this work highlights that timing is an important facet that needs consideration when examining pathogen evolution under a transmission–virulence trade-off. An important body of literature has examined the effect of timing of transmission and mortality in infected individuals on pathogen evolution, assuming that there is differential transmission and mortality throughout an infection before (potential) recovery (see e.g. [19,20] for theoretical developments, and [21] for connections with data). A major insight from the analysis of [18] is that as the timing of transmission and mortality decouple and mortality happens after transmission, there is strong selection for increased mortality with a classical trade-off between transmission and mortality. In related work, Osnas & Dobson [22] examined the evolution of virulence when the infected stage is partitioned into two parts, with potentially different transmission and disease-induced mortality (virulence) in each class. In the context of our study, while during-infection mortality can occur, PIM occurs after recovery when hosts can be (potentially) reinfected. Thus, the biological details of DIM and PIM differ starkly, not only on the timing of mortality but also on the characteristics of hosts while they are subject to such mortality. This key difference means that the previously infected susceptibles form a secondary ‘resource’ for the pathogen, which complicates evolutionary analyses even in the simplest settings. Furthermore, PIM has important epidemiological effects (see [6]), and its evolution could be affected by these changes in addition to other potential trade-offs. It is thus important to investigate the evolution of PIM.

PIM is also likely to be involved in a variety of trade-offs with other pathogen traits. In particular, a change in PIM could be accompanied by a change in either transmission, DIM, or immunity following recovery. For example, and in a similar manner to the classical trade-off between transmission and virulence [10], an increase in PIM could be driven by an increase in host damage during infection, itself caused by an increase in pathogen loads during infection. These elevated pathogen loads may also lead to increases in transmission or DIM. However, if the opposite relationship between viral loads and transmission occurs (e.g. because symptomatic hosts are too sick to have contacts), then an increase in PIM could lead to a decrease in transmission. Additionally, relative susceptibility to reinfection could vary with PIM, especially if they both are governed by within-host pathogen loads or the initial growth rate of pathogens after infection. It therefore seems likely that there will be correlations between PIM and other pathogen life-history traits. Thus, a complete understanding of the evolution of infectious disease requires elucidating the evolution of PIM.

To understand the eco-evolutionary dynamics of PIM, we use a general theoretical framework and investigate the evolutionary emergence of pathogens that cause PIM under a variety of potential trade-offs. We first extend the recently developed mathematical model of Saad-Roy *et al*. [6] (that includes susceptible buffering [23–26]) to include the invasion dynamics of an endemic pathogen that does not cause PIM by a mutant that does. Subsequently, we explore potential evolutionary effects when PIM modulates either the basic reproduction number only, relative susceptibility to reinfection only, or both of these features together. We then use our model framework to directly compare the evolution of PIM with that of DIM.

## 2. Model framework

The underlying epidemiological model we employ is as in Saad-Roy *et al.* [6] and is described as follows. Individuals are separated into three compartments: those that are fully susceptible ($S_{P}$), those that are currently infectious (I), and those that have recovered from infection and are (potentially partially) susceptible again ($S_{S}$). Furthermore, Λ denotes the constant recruitment rate, μ denotes the demographic mortality rate, β denotes the force of infection between infectious and fully susceptibles, γ denotes the recovery rate, $\alpha_{I}$ denotes the rate of DIM, ε denotes the relative susceptibility for a reinfection (where 0≤ε≤1), and $\alpha_{S}$ denotes the PIM rate. Finally, an important point to note is that, in our model, PIM occurs among individuals that are (potentially) susceptible to reinfection (instead of considering a second stage of infection where only mortality occurs, i.e. akin to a simplified version of that considered by Osnas & Dobson [22]). Note also that our model assumes no vertical transmission, and that the only post-infection impacts on hosts are on their mortality. The equations are given by


(2.1a) $$\begin{array}{ccc} & \frac{dS_{P}}{dt}=\Lambda -\beta S_{P}I-\mu S_{P}, & \end{array}$$

(2.1b) $$\begin{array}{ccc} & \frac{dI}{dt}=\beta I(S_{P}+\varepsilon S_{S})-(\gamma +\mu +\alpha_{I})I, & \end{array}$$

(2.1c) $$\begin{array}{ccc} & \frac{dS_{S}}{dt}=\gamma I-\varepsilon \beta S_{S}I-\mu S_{S}-\alpha_{S}S_{S}. & \end{array}$$

Since the total population is $N=S_{P}+I+S_{S}$, its rate of change is


(2.2) $$\begin{array}{ccc} & \frac{dN}{dt}=\Lambda -\mu N-\alpha_{I}I-\alpha_{S}S_{S}. & \end{array}$$

As shown in [6], it then follows that the basic reproduction number $R_{0}$ is


(2.3) $$\begin{array}{r} R_{0}=\frac{\beta \frac{\Lambda }{\mu }}{\gamma +\alpha_{I}+\mu }=\frac{\beta N_{0}}{\gamma +\alpha_{I}+\mu }, \end{array}$$

which is independent of PIM. Furthermore, Saad-Roy *et al.* [6] proved that this system has a unique endemic equilibrium $\hat{P}$ when $R_{0}>1$, and that if there is no PIM (i.e. $\alpha_{S}=0$), $\hat{P}$ is locally asymptotically stable. In theorem A.1 (Appendix), we extend this result to show that there exists δ>0 such that $\hat{P}$ is locally asymptotically stable for $0\leq \alpha_{S}<\delta$.

## 3. Results and discussion

**Figure 1.**
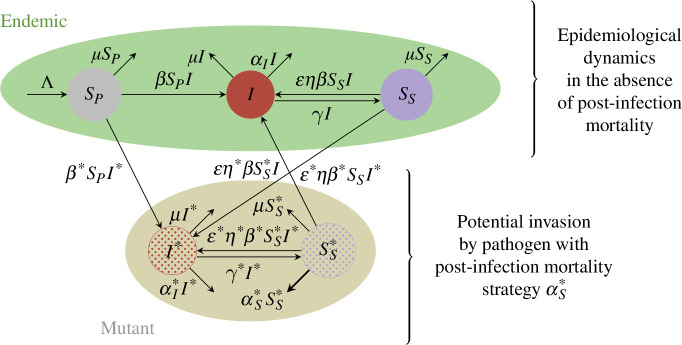
Schematic of the eco-evolutionary model for the evolution of PIM, with the epidemiological model (for the endemic pathogen) as in [6].

Taking the pathogen perspective, a key life-history question is whether any PIM can evolve (figure 1). Since the endemic equilibrium is locally stable for some interval of PIM $0\leq \alpha_{S}<\delta$ (δ>0), we assume the system is at $\alpha_{S}$, and we examine whether a mutant that causes a slightly different PIM can invade.

To perform this analysis, we consider the case where transmission, recovery, during-infection mortality, and relative susceptibility to reinfection may all depend on the value of PIM. Since we do not yet know the shapes of these dependences, and they will depend on the particular host–pathogen system, we keep them general and do not ascribe specific trade-off shapes. Thus, our results are broadly applicable across a range of systems. Furthermore, because we now have two pathogen strains (resident and mutant), the relative susceptibility to reinfection can depend on both the host and the pathogen. We separate this into its components and assume that ε and η denote the pathogen-component and host-component of relative susceptibility to reinfection, respectively. Thus, η denotes host-derived immunity to all pathogens, irrespective of their characteristics. For example, in the limiting case where η=0, a host has lifelong immunity against its infecting strain, but also against all possible other strains. Finally, to perform our evolutionary analysis, we assume that there is no superinfection, and that the mutant pathogen is rare.

The model equations for the endemic and mutant pathogen strategies, $\alpha_{S}$ and $\alpha_{S}^{*}$, respectively, are then given by


(3.1a) $$\begin{array}{ccc} & \frac{dS_{P}}{dt}=\Lambda -\beta S_{P}I-\beta^{*}S_{P}I^{*}-\mu S_{P}, & \end{array}$$

(3.1b) $$\begin{array}{ccc} & \frac{dI}{dt}=\beta I(S_{P}+\varepsilon \eta S_{S}+\varepsilon \eta^{*}S_{S}^{*})-(\gamma +\mu +\alpha_{I})I, & \end{array}$$

(3.1c) $$\begin{array}{ccc} & \frac{dS_{S}}{dt}=\gamma I-\varepsilon \eta \beta S_{S}I-\varepsilon^{*}\eta \beta^{*}S_{S}I^{*}-\mu S_{S}-\alpha_{S}S_{S}, & \end{array}$$

(3.1d) $$\begin{array}{ccc} & \frac{dI^{*}}{dt}=\beta^{*}I^{*}(S_{P}+\varepsilon^{*}\eta S_{S}+\varepsilon^{*}\eta^{*}S_{S}^{*})-(\gamma^{*}+\mu +\alpha_{I}^{*})I^{*}, & \end{array}$$

(3.1e) $$\begin{array}{ccc} & \frac{dS_{S}^{*}}{dt}=\gamma^{*}I^{*}-\varepsilon^{*}\eta^{*}\beta^{*}S_{S}^{*}I^{*}-\varepsilon \eta^{*}\beta S_{S}^{*}I-\mu S_{S}^{*}-\alpha_{S}^{*}S_{S}^{*}, & \end{array}$$

where $\varepsilon^{*}=\varepsilon [\alpha_{S}^{*}]$, $\eta^{*}=\eta [\alpha_{S}^{*}]$, $\beta^{*}=\beta [\alpha_{S}^{*}]$, $\alpha_{I}^{*}=\alpha_{I}[\alpha_{S}^{*}]$, $\gamma^{*}=\gamma [\alpha_{S}^{*}]$, $\varepsilon =\varepsilon [\alpha_{S}]$, $\eta =\eta [\alpha_{S}]$, $\beta =\beta [\alpha_{S}]$, $\alpha_{I}=\alpha_{I}[\alpha_{S}]$ and $\gamma =\gamma [\alpha_{S}]$. To summarize our mathematical framework, figure 1 depicts the invasion of an endemic pathogen that causes no PIM by a pathogen that does.

Computing the invasion reproduction number of a pathogen with strategy $\alpha_{S}^{*}$ (i.e. at the mutant-free equilibrium $\hat{P}$ with strategy $\alpha_{S}$; see [27]) gives that


(3.2) $$\begin{array}{rl} R_{E}[\alpha_{S},\alpha_{S}^{*}] & =\frac{\beta [\alpha_{S}^{*}]}{\gamma [\alpha_{S}^{*}]+\alpha_{I}[\alpha_{S}^{*}]+\mu }\left({\hat{S}}_{P}[\alpha_{S}]+\varepsilon [\alpha_{S}^{*}]\eta [\alpha_{S}]{\hat{S}}_{S}[\alpha_{S}]\right)\\ & =R_{0}[\alpha_{S}^{*}]\frac{\left({\hat{S}}_{P}[\alpha_{S}]+\varepsilon [\alpha_{S}^{*}]\eta [\alpha_{S}]{\hat{S}}_{S}[\alpha_{S}]\right)}{N_{0}}. \end{array}$$

Here, ${\hat{S}}_{P}$ and ${\hat{S}}_{S}$ are functions of $\alpha_{S}$ (directly, and also potentially indirectly via $\beta [\alpha_{S}]$, $\varepsilon [\alpha_{S}]$, $\eta [\alpha_{S}]$, $\gamma [\alpha_{S}]$, and $\alpha_{I}[\alpha_{S}]$).

Interestingly, note that the invasion reproduction number depends on PIM only indirectly, and the resident strategy $\alpha_{S}$ appears only via the endemic values of primary and secondary susceptibles. Thus, in contrast to the classic evolution of virulence (i.e. DIM), the evolutionary dynamics of PIM are completely determined by indirect effects. Since we are focused on pathogen characteristics, we assume in all that follows that $\eta [\alpha_{S}]=\eta [\alpha_{S}^{*}]=1$. Thus, the relative susceptibility to reinfection becomes entirely governed by its pathogen component.

### (a) Relative susceptibility to reinfection is independent of PIM

We begin our analyses by assuming that the relative susceptibility to reinfection is independent of PIM, i.e. $\varepsilon [\alpha_{S}]=\varepsilon_{0}$ with $0\leq \varepsilon_{0}\leq 1$. Since the model equations at equilibrium give that


(3.3) $$\begin{array}{ccc} & {\hat{S}}_{P}[\alpha_{S}]+\varepsilon_{0}{\hat{S}}_{S}[\alpha_{S}]=\frac{\gamma [\alpha_{S}]+\alpha_{I}[\alpha_{S}]+\mu }{\beta [\alpha_{S}]}, & \end{array}$$

it therefore follows that here the mutant invasion reproduction number is


(3.4) $$\begin{array}{r} R_{E}[\alpha_{S},\alpha_{S}^{*}]=\frac{R_{0}[\alpha_{S}^{*}]}{R_{0}[\alpha_{S}]}. \end{array}$$

Thus, $R_{E}[\alpha_{S},\alpha_{S}^{*}]$ is entirely determined by the mutant and resident one-strain basic reproduction numbers, which themselves do not depend separately on equilibrium numbers of either the never-infected or previously infected susceptibles. This surprising result emerges because the average susceptibility, which ‘coalesces’ both types of susceptibles together, is determined by $R_{0}$. Therefore, if $R_{0}[\alpha_{S}]$ is an increasing function of $\alpha_{S}$ at $\alpha_{S}=0$, (i.e. $R_{0}^{\prime }[0]>0$), then PIM is favoured evolutionarily (i.e. evolution toward $\alpha_{S}>0$). On the other hand, if $R_{0}[\alpha_{S}]$ is a decreasing function at $\alpha_{S}=0$, mutants with PIM cannot invade. (Note that these observations are reminiscent of those of Day [18] for the evolution of virulence under a transmission-mortality trade-off with different timing between transmission and mortality, where mortality after transmission favours higher virulence and mortality before transmission the opposite). Thus, if $R_{0}$ does not depend on PIM, i.e. transmission, recovery, and during-infection mortality are independent of PIM, then PIM is neutral.

### (b) Relative susceptibility to reinfection depends on PIM

While we previously examined the case where the relative susceptibility to reinfection following recovery and PIM are independent, these key mechanisms are likely very strongly related. For example, a pathogen that is capable of overwhelming immune responses in a previously infected host (i.e. high relative susceptibility to reinfection) may lead to high within-host pathogen loads, which could lead to substantial host damage and consequently increase PIM. In such a situation, relative susceptibility to reinfection increases with increases in PIM (i.e. $\varepsilon [\alpha_{S}]$ is an increasing function). On the other hand, a pathogen that is more successful at breaking through immune responses from prior infection may mean that the initial within-host pathogen growth rate is more limited (and thus the pathogen avoids detection). In such a scenario, the within-host damage incurred from infection may be milder, and the rate of PIM is lower. In this case, relative susceptibility to reinfection decreases with increasing PIM (i.e. $\varepsilon [\alpha_{S}]$ is a decreasing function).

If the relative susceptibility of reinfection varies depending on the degree of PIM, i.e. $\varepsilon [\alpha_{S}]$, then an adaptive dynamics approach (e.g. see [28–30]) is required to determine conditions that evolutionarily favour the emergence of PIM. In particular, these conditions hinge on the sign of the selection gradient $D[\alpha_{S}]=\frac{\partial R_{E}}{\partial \alpha_{S}^{*}}{|}_{\alpha_{S}^{*}=\alpha_{S}}$ at zero PIM. If D[0]>0, mutants with small, but positive, values of $\alpha_{S}$ can invade a resident with $\alpha_{S}=0$, and thus PIM is evolutionarily favoured to emerge. On the other hand, if D[0]<0, such mutants cannot invade a resident pathogen with no PIM, and therefore no PIM is an evolutionarily stable strategy (ESS).

#### (i) PIM only modulates relative susceptibility to reinfection

We first consider the simplifying example where transmission (β), recovery (γ) and DIM ($\alpha_{I}$) are independent of PIM, so that $R_{0}^{\prime }[\alpha_{S}]=0$. In this case, $D[\alpha_{S}]$ becomes


(3.5) $$\begin{array}{r} D[\alpha_{S}]=\varepsilon^{\prime }[\alpha_{S}]R_{0}[\alpha_{S}]\frac{{\hat{S}}_{S}[\alpha_{S}]}{N_{0}}. \end{array}$$

Therefore, it immediately follows from setting D[0]>0 that PIM is evolutionarily favoured if $\varepsilon^{\prime }[0]>0$, i.e. if the relative susceptibility to reinfection increases when PIM increases. On the other hand, no PIM is an ESS when $\varepsilon^{\prime }[0]<0$, i.e. if there is any ‘cost’ to an increase in PIM via a decrease in relative susceptibility to reinfection.

#### (ii) PIM impacts $R_{0}$ and relative susceptibility to reinfection

In the previous cases, we have examined the conditions that evolutionarily favour PIM if either only the basic reproduction number $R_{0}$ or (the pathogen-component of) relative susceptibility to reinfection ε depends on PIM ($\alpha_{S}$). However, since the underlying mechanisms that would give rise to each dependence could be closely related, both $R_{0}$ and ε could simultaneously be functions of $\alpha_{S}$. In this more general case, computing $D[\alpha_{S}]$ and using the equations at equilibrium gives that


(3.6) $$\begin{array}{r} D[\alpha_{S}]=\frac{R_{0}^{\prime }[\alpha_{S}]}{R_{0}[\alpha_{S}]}+\varepsilon^{\prime }[\alpha_{S}]R_{0}[\alpha_{S}]\frac{{\hat{S}}_{S}[\alpha_{S}]}{N_{0}}. \end{array}$$

Thus, PIM is evolutionarily favoured when D[0]>0, whereas no PIM is an ESS when D[0]<0. Rearranging D[0]>0 yields the condition that


(3.7) $$\begin{array}{r} \varepsilon^{\prime }[0]>-\frac{R_{0}^{\prime }[0]}{R_{0}[0{]}^{2}}\frac{N_{0}}{{\hat{S}}_{S}[0]}=\tau . \end{array}$$

We first consider the case where an increase in PIM leads to an increase in the single-strain basic reproduction number $R_{0}$, i.e. $R_{0}^{\prime }[0]>0$. For example, this occurs if transmission rises with increasing PIM, which is analogous the ‘classical’ transmission-virulence trade-off [31]. Alternatively, an increase in $R_{0}$ can also be due to a decrease in recovery or DIM with a larger PIM. More generally, we explore how the combination of these factors shapes the relationship between $R_{0}$ and PIM in remark A.1 (Appendix). In settings with $R_{0}^{\prime }[0]>0$, the threshold τ is negative, and PIM can be evolutionarily favoured even if its presence leads to a decrease in relative susceptibility to reinfection, i.e. when $0>\varepsilon^{\prime }[0]>\tau$. Thus, only a strong decrease in relative susceptibility to reinfection can select against PIM and prevent its emergence.

On the other hand, if $R_{0}^{\prime }[0]<0$, i.e. an increase in PIM leads to a decrease in the single-strain basic reproduction number, then τ>0 and no PIM can be an ESS even if the relative susceptibility to reinfection increases with PIM, i.e. when $0<\varepsilon^{\prime }[0]<\tau$. Here, the costs of PIM are considerable, and it is only evolutionarily favoured if it is accompanied with a strong increase in relative susceptibility to reinfection.

**Figure 2.**
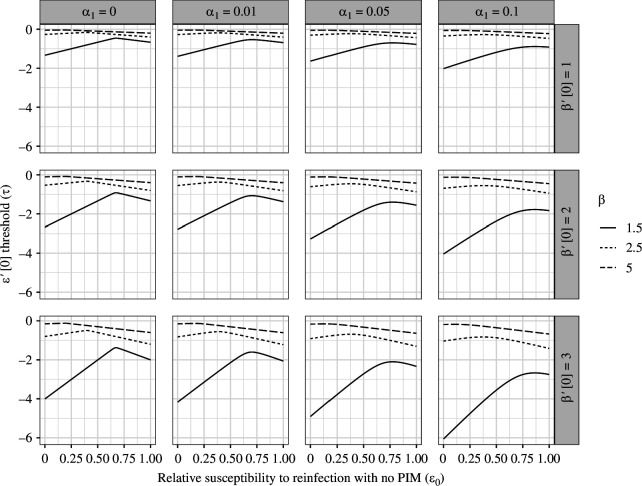
Conditions for the evolution of PIM as a function of without-PIM relative susceptibility to reinfection, assuming transmission increases with PIM. For values of $\varepsilon^{\prime }[0]$ above this threshold τ, mutants with PIM can invade. On the other hand, for values of $\varepsilon^{\prime }[0]$ below it, no PIM is an ESS. Other parameter values are $\Lambda =\mu =\frac{1}{50(52)}$ and γ=1.

In figure 2, we examine the characteristics of this threshold when transmission increases with PIM, as a function of the relative susceptibility to reinfection with no PIM, i.e. $\varepsilon_{0}=\varepsilon [0]$, across scenarios for DIM, transmission rate, and change of transmission with PIM (i.e. strength of the transmission-mortality trade-off). In certain settings, this threshold is clearly maximized at an intermediate value of $\varepsilon_{0}$. Thus, such intermediate values maximally constrain the emergence of PIM. Overall, as transmission increases, this threshold is closer to zero, and increasingly weaker increases in relative susceptibility to reinfection can prevent the evolutionary emergence of PIM. Finally, both during-infection mortality ($\alpha_{I}$) and the strength of the transmission-mortality trade-off ($\beta^{\prime }[0]$) also affect this threshold, with $\beta^{\prime }[0]$ having a more sizable effect.

**Figure 3.**
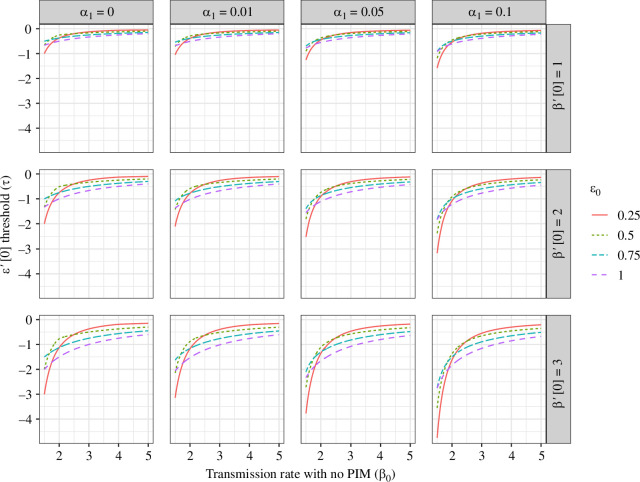
Condition for the evolution of PIM (i.e. the value of the threshold τ) as a function of the transmission rate with no PIM. In particular, for $\varepsilon^{\prime }[0]>\tau$ such that mutants with PIM can invade an endemic pathogen with no PIM, whereas $\varepsilon^{\prime }[0]<\tau$ implies no PIM is an ESS. The rows depict values of $\beta^{\prime }[0]$, i.e. the ‘initial' strength of the transmission–mortality trade-off, and the columns depict values of DIM. Note that other parameter values are as in figure 2.

In figure 3, we illustrate how τ varies with increasing transmission rates, for different rates of DIM and strengths of the relationship between transmission and mortality. As transmission increases, this threshold increases monotonically and approaches 0. Thus, for progressively higher transmission rates, increasingly moderate decreases in relative susceptibility to reinfection can select against the emergence of PIM.

### (c) Comparisons with disease-induced mortality during active infection

Since the pioneering work by Anderson and May [32], there have been a wealth of studies on the evolution of DIM (e.g. [10,31]), often referred to as ‘virulence’ ($\alpha_{I}$ in our model). Host characteristics when they experience DIM and PIM are fundamentally different. When PIM is possible, hosts have cleared the infection and may become reinfected as they are now in the pool of previously infected susceptible individuals (provided reinfection is possible), whereas when DIM is possible they are actively infected and are (by definition) not susceptible. A notable distinction between DIM and PIM is that, in the absence of any other relationship between model parameters, the basic reproduction number $R_{0}=\frac{\beta }{\gamma +\mu +\alpha_{I}}\frac{\Lambda }{\mu }$ is a decreasing function of DIM and independent of PIM.

This contrast between DIM and PIM has a number of consequences. Most importantly, theoretical models for pathogen evolution usually assume either a transmission-virulence trade-off, so that β is an increasing function of $\alpha_{I}$, or that recovery trades off with virulence, so that γ is a decreasing function of $\alpha_{I}$. In either of these cases, $R_{0}$ becomes a more complicated function of virulence, with potential non-monotonicies. On the other hand, if transmission or recovery trades off in a similar fashion with PIM (as described above for DIM), the basic reproduction number always increases for larger PIM (as noted earlier, this echoes the findings of Day [18] with respect to selection for different pathogen virulences based on the timing of mortality relative to that of transmission).

To examine the evolutionary implications of the differences between PIM and DIM, we set $\alpha_{S}=0$ in our model framework (Eq. [3.1]), and we perform equivalent calculations with $\alpha_{I}$ instead of $\alpha_{S}$. If the relative susceptibility to reinfection is independent of DIM, i.e. $\varepsilon [\alpha_{I}]=\varepsilon_{0}$, it follows as previously that the mutant invasion reproduction number is simply the ratio of the one-strain basic reproduction numbers of the invading pathogen to that resident, i.e.


(3.8) $$\begin{array}{r} R_{E}[\alpha_{I},\alpha_{I}^{*}]=\frac{R_{0}[\alpha_{I}^{*}]}{R_{0}[\alpha_{I}]}, \end{array}$$

which is qualitatively similar to that with PIM (except that $R_{0}[\alpha_{I}]$ and $R_{0}[\alpha_{I}^{*}]$ depend directly on $\alpha_{I}$ and $\alpha_{I}^{*}$, respectively).

On the other hand, if relative susceptibility to reinfection depends on DIM, further (analogous) analyses yield that the selection gradient D for DIM is


(3.8) $$\begin{array}{r} D[\alpha_{I}]=\frac{R_{0}^{\prime }[\alpha_{I}]}{R_{0}[\alpha_{I}]}+\varepsilon^{\prime }[\alpha_{I}]\frac{{\hat{S}}_{S}[\alpha_{I}]}{N_{0}}. \end{array}$$

The first key observation is that in general $R_{0}^{\prime }[\alpha_{I}]\neq 0$ even if transmission and recovery of independent of $\alpha_{I}$, unless the transmission rate perfectly scales the inverse of the average duration of infection (i.e. $\beta \propto \mu +\gamma +\alpha_{I}$). Thus, rearranging D[0]>0 gives that


(3.9) $$\begin{array}{r} \varepsilon^{\prime }[0]>-\frac{R_{0}^{\prime }[0]}{R_{0}[0{]}^{2}}\frac{N_{0}}{{\hat{S}}_{S}[0]}, \end{array}$$

whereas D[0]<0 when $\varepsilon^{\prime }\left[0\right]<-\frac{R_{0}^{\prime }\left[0\right]}{R_{0}{\left[0\right]}^{2}}\frac{N_{0}}{{\hat{S}}_{S}\left[0\right]}.$

Suppose that DIM only modulates relative susceptibility to reinfection (i.e. transmission and recovery are independent of $\alpha_{I}$), and that relative susceptibility to reinfection increases with increasing mortality ($\varepsilon^{\prime }[0]>0)$, i.e. there is a susceptibility-mortality trade-off. Therefore, $R_{0}^{\prime }[0]=-\frac{\beta N_{0}}{(\gamma +\mu {)}^{2}}<0$, and thus no DIM is evolutionarily favoured for $0<\varepsilon^{\prime }[0]<-\frac{R_{0}^{\prime }[0]}{R_{0}[0{]}^{2}}\frac{N_{0}}{{\hat{S}}_{S}[0]}$. Under the same conditions, we previously showed that PIM was evolutionarily favoured (see equation (3.5) and discussion afterwards). Thus, a key result is that pathogens can more easily evolve PIM than DIM because of lower costs.

More generally, our model allows us to directly compare the threshold for the susceptibility-mortality relationship (evaluated at no mortality) that selects for DIM or PIM. Here, we consider both settings where there is no additional mortality, and we investigate the conditions for which either source of mortality is evolutionarily favoured. If transmission or recovery trades off with $\alpha_{I}$, this threshold is


(3.10) $$\begin{array}{r} \varepsilon^{\prime }[0]>-\frac{\beta^{\prime }[0](\gamma [0]+\mu )-\beta [0](\gamma^{\prime }[0]+1)}{\beta [0{]}^{2}{\hat{S}}_{S}[0]}=\tau_{\alpha_{I}}, \end{array}$$

whereas the equivalent condition for PIM is


(3.11) $$\begin{array}{r} \varepsilon^{\prime }[0]>-\frac{\beta^{\prime }[0](\gamma [0]+\mu )-\beta [0]\gamma^{\prime }[0]}{\beta [0{]}^{2}{\hat{S}}_{S}[0]}=\tau_{\alpha_{S}}. \end{array}$$

Most interestingly, if


(3.12) $$\begin{array}{r} \frac{\beta [0]}{\gamma [0]+\mu }\gamma^{\prime }[0]<\beta^{\prime }[0]<\frac{\beta [0]}{\gamma [0]+\mu }\left(1+\gamma^{\prime }[0]\right), \end{array}$$

it can be seen that $\tau_{\alpha_{I}}>0$ but $\tau_{\alpha_{S}}<0$. Thus, in this setting, an important difference between the evolution of DIM and that of PIM emerges. From the perspective of the pathogen, a susceptibility–mortality trade-off (i.e. $\varepsilon^{\prime }[0]>0$) can select against DIM but does not limit the evolution of PIM. In particular, for DIM to be selected for in this setting, it must lead to a strong enough increase in relative susceptibility to reinfection. For increases in relative susceptibility to reinfection with $0<\varepsilon^{\prime }[0]<\tau_{\alpha_{I}}$, DIM is selected against, and no DIM is an ESS. On the other hand, a strategy with PIM can invade if it is accompanied by a decrease in relative susceptibility to reinfection, as long as this change is not too drastic.

**Figure 4.**
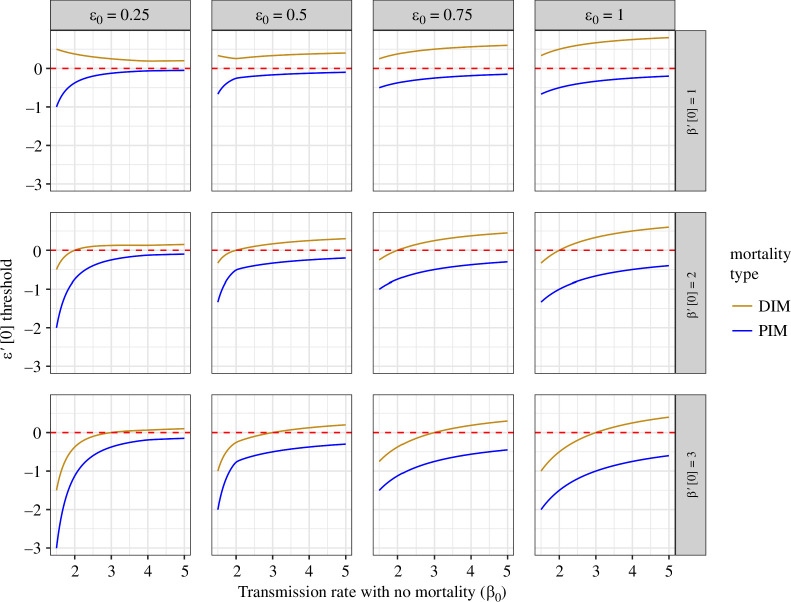
Comparison of the evolution of DIM and PIM under a relationship between relative susceptibility to reinfection and mortality, when transmission increases with mortality (i.e. $\beta^{\prime }[0]>0$). The rows denote different scenarios for the change in transmission with mortality, and the columns are different scenarios of baseline relative susceptibility to reinfection without mortality. As before, for values of $\varepsilon^{\prime }[0]$ above each curve, mutants with mortality can invade, whereas no mortality is an ESS for values of $\varepsilon^{\prime }[0]$ beneath them. Below the dashed red line at $\varepsilon^{\prime }[0]=0$, a mutant with either PIM or DIM pays a cost as it elicits lower relative susceptibility to reinfection. On the other hand, above this line, a mutant with either PIM or DIM elicits higher relative susceptibility to reinfection. Note that for PIM evolution, we set $\alpha_{I}=0$, and for DIM evolution, we set $\alpha_{S}=0$, and other parameter values are as in figure 2.

In figure 4, we illustrate these necessary thresholds $\tau_{\alpha_{I}}$ and $\tau_{\alpha_{S}}$, which lead to selection for either DIM or PIM, respectively, as a function of the no-mortality transmission rate ($\beta_{0}$). We also examine this in different contexts, with a range of initial values for both the relative susceptibility to reinfection ($\varepsilon_{0}$) and a transmission–mortality trade-off ($\beta^{\prime }[0]$). When $\varepsilon_{0}$ and $\beta^{\prime }[0]$ are both small, the relationship between $\varepsilon^{\prime }[0]$ and $\beta_{0}$ for DIM and PIM can be fundamentally different. In particular, an increase in no-mortality transmission can decrease the threshold necessary for the evolution of DIM, whereas an increase in this transmission rate always increases the threshold for PIM. Furthermore, when the transmission-mortality trade-off is weak (i.e. small $\beta^{\prime }[0]$), the thresholds for DIM are always positive. Intuitively, in this case, the transmission advantages to the pathogen of an increased DIM are moderate, and thus DIM is selected against unless it is accompanied by a strong increase in relative susceptibility to reinfection. As $\beta^{\prime }[0]$ increases, pathogen benefits of DIM increase, which enables DIM to be selected for under lower thresholds of $\varepsilon^{\prime }[0]$.

### (d) Caveats and future directions

To examine the qualitative contexts that surround the evolution of PIM, we have used a simple mathematical framework with a number of simplifying assumptions. First, to focus on the evolution of PIM, we have examined whether mutants that cause PIM can invade a resident pathogen that causes none. However, in cases where PIM is selected for, we have ignored the longer-term evolutionary outcomes of this strategy. In particular, it would be valuable in future work to determine where nonzero ESS(s) for PIM are located, and how they are influenced by the shapes of various underlying trade-offs. Furthermore, since elevated PIM can cause epidemiological periodicity, understanding the effects of these cycles on the evolutionary dynamics of PIM is also an important area of research.

While our work is theoretical, a major future direction of research is to empirically test our predictions in experimental host–pathogen systems. For example, in an empirical SIS system, experiments should first examine whether previously infected hosts exhibit PIM. If they do, further studies should examine the evolution of PIM, and the underlying trade-offs that govern it, more closely. In particular, while we have used a theoretical framework to study the effect of general dependences among life-history traits on the evolution of PIM, determining which of these trade-offs occur empirically, and their shapes, would be extremely valuable. In a related, but different, empirical direction, understanding the evolution of PIM for circulating human pathogens would also be important. For example, examining the rates of post-infection symptoms (and their potential effects on mortality) for different SARS-CoV-2 variants (or for other evolving endemic pathogens) may be valuable.

We have also assumed that there are no differences between primary infections and reinfections. In reality, PIM could increase due to prolonged damage of multiple infections, and immunity could accumulate or reduce with subsequent exposures (see e.g. discussions in [24,33]). Thus, an important avenue of future research is to incorporate multiple infection classes, each leading to their own rates of PIM and relative susceptibility to subsequent infections. Relatedly, we have ignored a period of ‘perfect’ immunity where individuals cannot get infected (e.g. akin to the general models of [23,24]) and have instead focused on the case where individuals recover and are immediately potentially susceptible again. Such a period of complete immunity could impact both the epidemiological dynamics with PIM and its evolution, and it would be valuable to examine this further in future work.

In our model, we have also omitted vaccination. However, the deployment of vaccines can have important effects on epidemiological dynamics (e.g. [24,25,34]). Furthermore, vaccination could have impacts on evolutionary dynamics, and, a number of studies have examined this in various contexts, see e.g. [35–40]. Therefore, a salient area of future development is to examine the evolution of PIM in the presence of vaccination. Relatedly, therapeutic treatments, such as antivirals (for viruses), could decrease within-host pathogen damage, and in turn decrease rates of DIM and PIM. In parallel, a large body of work has shown that these therapeutics can affect evolutionary trajectories (see e.g. [41]), and [42] for a COVID-19-specific example) and can catalyse important changes (e.g. evolution of drug resistance [43]). While we have ignored therapeutics and their potential evolutionary effects on pathogen-caused mortality, refinements of our framework should be used to examine these questions.

Furthermore, we have assumed a homogeneous population and ignored heterogeneity in our model. Previous work has examined various sources of heterogeneity, including in space [12,44,45] or in disease characteristics (e.g. transmission [46,47] or age [48]), and shown that these can have important effects on evolutionary and epidemiological trajectories. Thus, including these features in our eco-evolutionary model framework is an important future direction. Relatedly, we have focused on population-level processes and omitted explicit within-host kinetics (see e.g. [14]). However, mechanisms that affect rates of PIM and DIM, relative susceptibility to reinfection, and transmission are intricate processes within hosts. Thus, it would be valuable to develop appropriate within-host model characteristics and couple these to our population-level framework.

Finally, we have ignored the dynamics of human behaviour (e.g. [49]), and these could hinge on DIM and PIM. In particular, changes in DIM or PIM could affect perceived individual risks from infection. For example, individuals who perceive high risk of DIM or PIM may alter their behaviour to decrease their likelihood of infection. On the other hand, if DIM or PIM is low, individuals may not view an infection as risky and thus may be less willing to adhere to nonpharmaceutical interventions. In turn, these behavioural effects could shape epidemiological dynamics, which could themselves influence evolutionary trajectories. To untangle these effects, it would be useful to develop novel epidemiological models that incorporate social dynamics (see e.g. [50,51]) that include DIM and PIM in the underlying framework. These cross-scale models could then be used to study the evolution of PIM and its interplay with behavioural-epidemiological dynamics.

## 4. Conclusions

The COVID-19 pandemic has underscored the effects of infections on their hosts long after recovery. These complications can exhibit themselves through a constellation of symptoms, which include elevated mortality (either directly, or as an indirect consequence of other effects). While such PIM may be a general characteristic of many pathogens, it has so far been generally overlooked in infectious disease eco-evolutionary dynamics. However, the potential burden that PIM poses on host populations may be considerable, especially for endemic pathogens. Thus, understanding the life history strategy of this trait in various settings is necessary for proper disease management.

In this article, we addressed this gap and resolved the eco-evolutionary dynamics of PIM emergence. To accomplish this, we used a recent epidemiological model that separates never-infected susceptibles from those that were previously infected (and potentially subject to PIM) and performed evolutionary analyses. Under a variety of settings, we have shown that PIM can readily evolve. Surprisingly, while pre- and post-infection susceptibles are two ‘resources’ for the pathogen from an ecological standpoint, if the relative susceptibility to reinfection is independent of PIM, we find that the one-strain basic reproduction number $R_{0}$ governs the evolution of PIM. In settings where relative susceptibility to reinfection changes with PIM, we have derived conditions on this relationship that guarantee that either zero PIM is an ESS, or that PIM is evolutionarily favoured. If $R_{0}$ is independent of PIM, then any trade-off between relative susceptibility to reinfection and mortality selects against PIM. This condition is relaxed when other disease parameters, such as transmission or DIM, also depend on the rate of PIM, and, crucially, lead to an increase in $R_{0}$ with PIM. In this setting, PIM can evolve even if it leads to a (moderate) decrease in relative susceptibility to reinfection (very rapid decreases select against PIM).

Finally, we have contrasted the evolution of PIM with that of DIM. The key insight of this latter analysis is that, in otherwise identical settings, PIM can more easily evolve than DIM. This result can be traced to the direct dependence of $R_{0}$ on DIM, which imparts an immediate cost of an increase in DIM to pathogen fitness. Overall, our findings highlight the ease with which pathogens capable of causing PIM can be selected for. In particular, our theoretical predictions provide testable hypotheses, which yield themselves to further experimental investigation in controlled laboratory host–pathogen systems. Thus, given the potential widespread impact of PIM on populations, future studies on pathogen-specific evolutionary ecology should examine this characteristic in detail. In tandem, our work also stresses the importance of measuring current rates of PIM, especially for circulating pathogens, such as SARS-CoV-2. More generally, to characterize PIM and its evolutionary trajectories, large immuno-epidemiological cohort studies are needed (see [52]).

## Data Availability

Code to reproduce the figures is available as electronic supplementary material [53].

## Appendix

**Theorem A.1**. *When it exists, the endemic equilibrium* $\hat{P}$ *is locally asymptotically stable at least for* $\alpha_{S}$ *in a neighbourhood of 0, i.e. there exists* δ>0 *such that* $\hat{P}$ *is locally asymptotically stable for at least* $0\leq \alpha_{S}<\delta$*.*

*Proof. Taking the equations for the epidemiological model (using equations* (2.1c*), (2.1b) and (2.2) from the main text) the dynamics are as follows:*

$$\begin{array}{c} \frac{dS_{S}}{dt}=\gamma I-\varepsilon \beta S_{S}I-(\alpha_{S}+\mu )S_{S}, \end{array}$$

$$\begin{array}{c} \frac{dI}{dt}=\beta [(N-S_{S}-I)+\varepsilon S_{S}]I-(\mu +\gamma +\alpha_{I})I, \end{array}$$

$$\begin{array}{c} \frac{dN}{dt}=\Lambda -\mu N-\alpha_{I}I-\alpha_{S}S_{S}, \end{array}$$

Stability can be determined by calculating the eigenvalues of the Jacobian, J, at the endemic steady state, $\hat{P}=({\hat{S}}_{S},\hat{I},\hat{N})$, which is positive when it exists (i.e. $R_{0}>1$, see [6]), where

$$\begin{array}{c} J(\hat{P})=\left(\begin{array}{ccc} -\varepsilon \beta \hat{I}-(\alpha_{S}+\mu ) & \gamma -\varepsilon \beta {\hat{S}}_{S} & 0\\ -(1-\varepsilon )\beta \hat{I} & -\beta \hat{I} & \beta \hat{I}\\ -\alpha_{S} & -\alpha_{I} & -\mu \end{array}\right). \end{array}$$

First, note that to show that the eigenvalues of a 3×3 matrix, A, have negative real part, it suffices to check the Routh-Hurwitz conditions, i.e. that tr(A)<0, det⁡(A)<0, and $\text{tr}(A)a_{2}-\det(A)<0$, where $a_{2}$ denotes the sum of the principal minors of A.

Since $\gamma -\varepsilon \beta {\hat{S}}_{S}=(\alpha_{S}+\mu )\frac{{\hat{S}}_{S}}{\hat{I}}$, it follows that

$$\begin{array}{c} J(\hat{P})=\left(\begin{array}{ccc} -\varepsilon \beta \hat{I}-(\alpha_{S}+\mu ) & (\alpha_{S}+\mu )\frac{{\hat{S}}_{S}}{\hat{I}} & 0\\ -(1-\varepsilon )\beta \hat{I} & -\beta \hat{I} & \beta \hat{I}\\ -\alpha_{S} & -\alpha_{I} & -\mu \end{array}\right). \end{array}$$

Therefore,

$$\begin{array}{r} \text{tr}(J(\hat{P}))=-(\varepsilon \beta \hat{I}+\alpha_{S}+\mu +\beta \hat{I}+\mu )<0. \end{array}$$

Computing the determinant across the third column gives

$$\begin{array}{rl} \det(J(\hat{P})) & =-\mu [(\varepsilon \beta \hat{I}+\alpha_{S}+\mu )\beta \hat{I}+(1-\varepsilon )\beta {\hat{S}}_{S}(\alpha_{S}+\mu )]-\beta \hat{I}\left[(\varepsilon \beta \hat{I}+\alpha_{S}+\mu )\alpha_{I}+\alpha_{S}(\alpha_{S}+\mu )\frac{{\hat{S}}_{S}}{\hat{I}}\right]\\ & =-(\alpha_{I}+\mu )(\varepsilon \beta \hat{I}+\alpha_{S}+\mu )\beta \hat{I}-\beta {\hat{S}}_{S}(\alpha_{S}+\mu )[\mu (1-\varepsilon )+\alpha_{S}]<0. \end{array}$$

Finally, computing $a_{2}$ gives

$$\begin{array}{rl} a_{2} & =\left|\begin{array}{cc} -\varepsilon \beta \hat{I}-(\alpha_{S}+\mu ) & (\alpha_{S}+\mu )\frac{{\hat{S}}_{S}}{\hat{I}}\\ -(1-\varepsilon )\beta \hat{I} & -\beta \hat{I} \end{array}\right|+\left|\begin{array}{cc} -\varepsilon \beta \hat{I}-(\alpha_{S}+\mu ) & 0\\ -\alpha_{S} & -\mu \end{array}\right|+\left|\begin{array}{cc} -\beta \hat{I} & \beta \hat{I}\\ -\alpha_{I} & -\mu \end{array}\right|\\ & =(\beta \hat{I}+\mu )(\varepsilon \beta \hat{I}+\alpha_{S}+\mu )+(\mu +\alpha_{I})\beta \hat{I}+(1-\varepsilon )\beta {\hat{S}}_{S}(\alpha_{S}+\mu ). \end{array}$$

Thus, the third condition can be written as

$$\begin{array}{rl} tr(J(\hat{P}))a_{2}-\det(J(\hat{P})) & =-(\beta \hat{I}+\mu )(\varepsilon \beta \hat{I}+\alpha_{S}+\mu {)}^{2}-(\beta \hat{I}+\mu {)}^{2}(\varepsilon \beta \hat{I}+\alpha_{S}+\mu )-(\mu +\alpha_{I})\beta \hat{I}(\beta \hat{I}+\mu )\\ & \;\;\;-\beta {\hat{S}}_{S}(\alpha_{S}+\mu )[(1-\varepsilon )\varepsilon \beta \hat{I}+(1-\varepsilon )\beta \hat{I}+(1-\varepsilon )\mu -\varepsilon \alpha_{S}]. \end{array}$$

If $\alpha_{S}=0$, it can be seen (and is shown in [6]) that $\text{tr}(J(\hat{P}))a_{2}-\det(J(\hat{P}))<0$. Therefore, for positive values of $\alpha_{S}$, either $\text{tr}(J(\hat{P}))a_{2}-\det(J(\hat{P}))<0$, and $\hat{P}$ is locally asymptotically stable. Or as $\alpha_{S}$ increases we may have a switch such that $\text{tr}(J(\hat{P}))a_{2}-\det(J(\hat{P}))>0$, but if this occurs, then, by continuity, there is a value δ>0 such that $\text{tr}(J(\hat{P}))a_{2}-\det(J(\hat{P}))<0$ for $0\leq \alpha_{S}<\delta$, which completes the proof.∎

**Remark A.1**. *Taking the first derivative of* $R_{0}[\alpha_{S}]$ *and evaluating it at* $\alpha_{S}=0$ *gives that* $R_{0}^{\prime }[0]>0$ if

(A.1)
$$\begin{array}{ccc} & \beta^{\prime }[0]>\frac{\beta [0]}{\gamma [0]+\alpha_{I}[0]+\mu }\left(\gamma^{\prime }[0]+\alpha_{I}^{\prime }[0]\right), & \end{array}$$

*whereas* $R_{0}^{\prime }[0]<0$ if

(A.2)
$$\begin{array}{ccc} & \beta^{\prime }[0]<\frac{\beta [0]}{\gamma [0]+\alpha_{I}[0]+\mu }\left(\gamma^{\prime }[0]+\alpha_{I}^{\prime }[0]\right). & \end{array}$$
